# Multiple Genetic Alterations as Resistance Mechanism during Second-Line Lorlatinib for Advanced ALK-Rearranged Lung Adenocarcinoma: A Case Report

**DOI:** 10.3390/diagnostics12030682

**Published:** 2022-03-11

**Authors:** Annamaria Catino, Rosanna Lacalamita, Simona De Summa, Francesco Pesola, Stefania Tommasi, Domenico Galetta

**Affiliations:** 1 Thoracic Oncology Unit, IRCCS Istituto Tumori “Giovanni Paolo II”, Viale Orazio Flacco 65, 70124 Bari, Italy; f.pesola@oncologico.bari.it (F.P.); galetta@oncologico.bari.it (D.G.); 2 Molecular Diagnostics and Pharmacogenetics Unit, IRCCS Istituto Tumori “Giovanni Paolo II”, Viale Orazio Flacco 65, 70124 Bari, Italy; r.lacalamita@oncologico.bari.it (R.L.); desumma.simona@gmail.com (S.D.S.); s.tommasi@oncologico.bari.it (S.T.)

**Keywords:** ALK-rearranged lung cancer, resistance mechanisms, lorlatinib, case report

## Abstract

Second and third-generation ALK-TKI inhibitors have showed better activity and have replaced crizotinib in most of cases of advanced ALK-rearranged lung adenocarcinoma. The emergence of resistance adversely affects also the activity of these newer drugs; in particular, lorlatinib often shows multiple and complex resistance mechanisms. The case reported here highlights the importance of reassessing the biomolecular profile during the disease course, both by tissutal and liquid biopsy, with the aim of improving the knowledge of these resistance mechanisms, and so identifying new drugs or sequences able to optimize the management of these patients.

## 1. Introduction

Lung adenocarcinoma harbors anaplastic lymphoma kinase (ALK) rearrangements in about 3% to 5% of cases in western countries.

Mostly detected in young and never smokers patients, ALK rearrangements occur frequently with the EML4 gene as fusion partner. Even though the clinical significance of the different fusion transcripts is still undefined, E13;A20 and E6a/b;A20 are the most common variants of EML4-ALK, accounting for approximately 33% and 29% of cases, respectively [1].

Until recently, the ALK-tyrosine kinase inhibitor (ALK-TKI) crizotinib has represented the standard first-line treatment in advanced lung adenocarcinoma patients with this genetic alteration, following the results of previous studies that showed the superior effectiveness both in response rate and survival as compared to chemotherapy [2].

More recently, second-generation ALK-TKIs have demonstrated better activity, including against brain metastases, and very good tolerability, having therefore replaced crizotinib in most cases [3,4,5,6,7], both in first-line treatment and in patients with acquired resistance to crizotinib.

However, the efficacy of first and second generation ALK inhibitors can be limited by the development of acquired resistance mechanisms that emerge during treatment [8,9]. Various resistance mechanisms have been detected: ALK-dependent mutations, ALK-amplifications, and ALK-independent mechanisms [10,11]. Among these, point mutations in the kinase domain such as L1196M, L1152R, C1156Y, G1202R, G1269A, S1206Y, F1174C, and 1151Tins have been described and characterized.

Lorlatinib, a third generation ALK/ROS-TKI, has shown efficacy in patients who have progressed under second-generation ALK-TKIs and against most ALK resistance mutation as G1202R, which is considered the most common resistance mutation to alectinib; in addition, lorlatinib is able to better overcome the blood–brain barrier [8].

Moreover, the occurrence of multiple concomitant mutations during TKI treatment has been hypothesized as a possible synergistic mechanism that mediates resistance to both second and third generation TKIs; hence, the molecular characterization of resistant tumors may represent the basis for the development of new drugs able to overcome resistance and finally to improve the therapeutic strategy [12].

Targeted RNA and DNA next generation sequencing (NGS) is the suggested methodology to evaluate ALK alterations both in tissue and in plasma. NGS allows parallel sequencing of multiple sites of the ALK gene and of multiple fusion transcripts at a very high depth of coverage. The use of NGS in real life diagnostics allows to detect alterations of drug sensitivity and/or drug resistance in the same run, so decreasing turnaround time and costs.

Herein, we describe the dismal clinical outcome of a patient acquiring multiple ALK mutations after lorlatinib treatment; we also highlight the importance of molecular assessment both by tissue and liquid biopsy NGS during treatment in order to provide information about the often complex and various acquired resistance mechanisms to ALK-inhibitors, particularly to lorlatinib, given the predictably increasing widespread use of this drug. 

This case report was prepared following the CARE Guidelines [13].

## 2. Case Presentation

In March 2019, a 71-year-old woman, light former smoker (7 pack/year) was referred to our institution due to the diagnosis of metastatic lung adenocarcinoma (stage IV due to brain metastases, pleural infiltration with effusion, and ilar-mediastinal plus supraclavicular lymph node involvement). 

Molecular analysis of the pleural biopsy detected an ALK rearrangement with a strong positivity by immunohistochemistry (Ventana ALK D5F3 CDx assay), so the patient started on alectinib, obtaining a marked clinical and radiological response lasting eight months until October 2019.

In November 2019, due to disease progression both in the same initial sites and also at the abdominal lymph nodes and contralateral pleura, the patient underwent molecular profiling sequencing plasma cell free DNA (cfDNA) by a target panel of >320 genes able to detect substitutions, insertion and deletion alterations, copy number alterations, and gene rearrangements (FoundationOne^®^Liquid). Such a large panel, analyzed in outsourcing, was used to investigate potential alterations of resistance in ALK and in other genes trying to overcome intra-tumor heterogeneity. The NGS study confirmed the EML4-ALK fusion variant 3a/b (fusion points E6a;A20) and revealed the presence of ALK mutation G1202R. 

The patient started treatment with the third generation ALK-TKI lorlatinib, obtaining a clinical improvement and a radiological partial response in all disease sites. After 3 months, due to disease progression with right pleural effusion, lung ilar nodes, pericardial effusion, and bone metastases, the patient was submitted to pleural drainage and biomolecular reassessment. The NGS assay was performed in-house on cell blocks of pleural effusions (Oncomine Solid Tumor DNA—Thermofisher), showing three concomitant ALK mutations. The alteration c. 3604G > A; p.G1202R of resistance to the first-generation treatment was still present (allelic frequency (AF) 40.5%), and the c.3617C > T p.S1206F alteration (AF 20.7%), which appeared to be in *cis*; c.3452C > A p.T1151K with AF 14%, also occurred. A course of chemotherapy with carboplatin plus pemetrexed was administered without any clinical improvement, and the patient died in March 2020.

**Figure 1:**
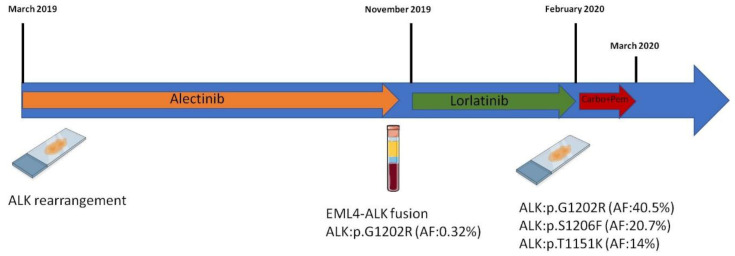
Timeline of the reported case.

The timeline of the clinical course of the case is shown in Figure 1. 

## 3. Discussion

In the reported case, the disease progression during alectinib therapy was characterized by the persistence of ALK translocation together with the occurrence of the point mutation G1202R, both detected by liquid biopsy.

Although lorlatinib is the standard treatment after progression from alectinib and generally also chosen irrespective of a new biomolecular profiling, in this case, the genetic reassessment by NGS was performed before starting lorlatinib with the aim of identifying and studying any new possible lorlatinib resistance mutations. This choice was mainly based on academic purposes, since to re-test the disease during the clinical course in oncogene-addicted patients could be helpful both to obtain biological data about these still little-known resistance mechanisms and also to select biomolecular profiles with worse prognosis deserving closer monitoring in clinical practice.

Moreover, a crucial issue of the case is that the resistance to lorlatinib occurred after only three months from the initial clinical response. The NGS analysis revealed the presence of p.G1202R along with other two mutations in the ALK gene (p.S1206F and p.T1151K). 

The possibility that the acquired resistance to lorlatinib can be mediated by the acquisition of multiple and different compound ALK mutations but also by histological small cell transformation is supported by recent studies that have underscored the still scarce knowledge on ALK TKIs issue due to the heterogeneity of this mechanism [12,14].

Uncommon acquired resistance mutations to crizotinib can also lead to double mutations when subsequently treated with lorlatinib [15]; furthermore, the possible and frequent onset of compound ALK mutations may confer lorlatinib resistance at the time of progression under crizotinib, alectinib, and ceritinib. In addition, the presence of the EML4-ALK fusion variant 3 and/or TP53 mutations identifies cases with worse clinical outcome.

Hence, plasma-based next-generation sequencing (NGS) is increasingly used in clinical practice, due to its feasibility and usefulness of serial ctDNA assays during the clinical course, to provide data and reassess the molecular profile, including the onset of resistance mechanisms, so as to better monitor and optimize the treatment [12,16,17,18,19,20].

The p.G1202R/p.S1206F has been previously described as a lorlatinib compound resistance mutation [21], while p.T1151K has been described as a resistance mutation to crizotinib and alectinib [22,23] but not to lorlatinib. 

Furthermore, in some cases, the possible resensitization of lorlatinib-resistant compound mutations to first- or second-generation ALK-TKIs has been described, suggesting to re-purpose some drugs [24].

Finally, as a further challenging issue that makes the scenario more complex, the clinical outcome of our patient confirms literature data reporting a possible lower responsivity of ALK-rearranged patients, previously treated with second-generation ALK-TKI inhibitors, to the standard platinum plus pemetrexed combination [25].

Thus, in these patients, the choice of chemotherapy should be carefully evaluated, due to the not fully predictable response to platinum–pemetrexed-based regimens.

In the reported case, the unsatisfactory response to lorlatinib as second-line after second generation TKI was rather unexpected if we take into account the data derived from previously published studies [26,27]; therefore, it could be due to intratumor heterogeneity and other mechanisms, including bypass signaling, the epithelial-to-mesenchymal transition, and phenotypical changes.

Unfortunately, at the time of the last progression, the patient’s clinical status did not allow for further diagnostic procedures to reassess the disease profile. 

In order to understand the effects of the three mutations on the ALK protein structure, a computational study was conducted. 

**Figure 2:**
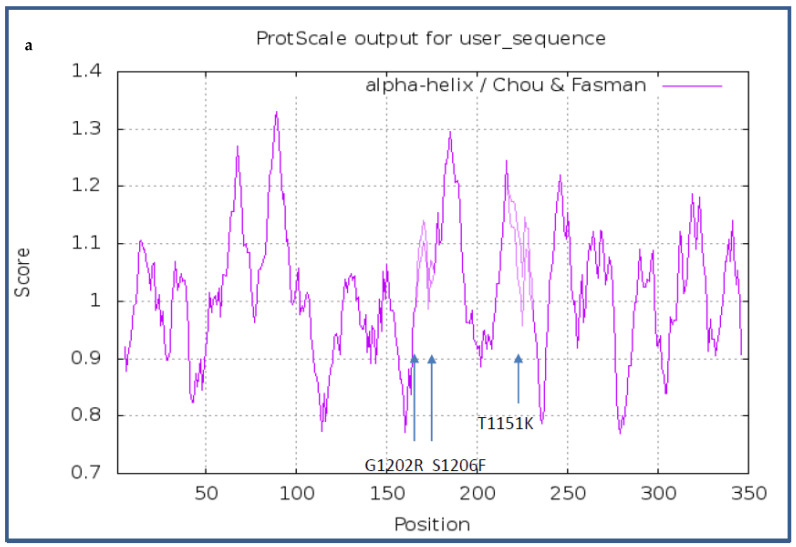

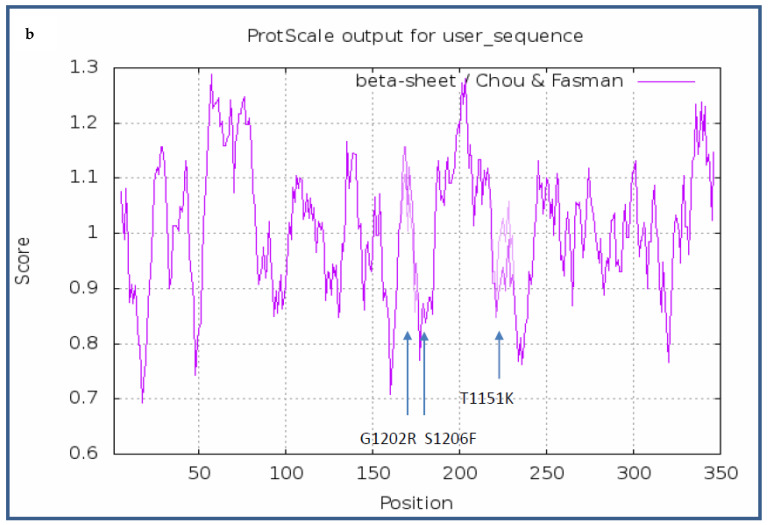
(**a**,**b**) Results of the prediction of the Chou and Fasman algorithm for (**a**) alpha-helix and (**b**) beta-sheet. A decrease was observed in the propensity score at the position of the three alterations.

The Chou and Fasman algorithm [28] was applied to the protein primary sequence to verify the effect on secondary structures, in particular alpha-helix and beta-sheet. As shown in Figure 2, all three ALK alterations showed a difference in the propensity scores both for alpha-helix and beta-sheet when compared to the wild-type sequence.

**Figure 3:**
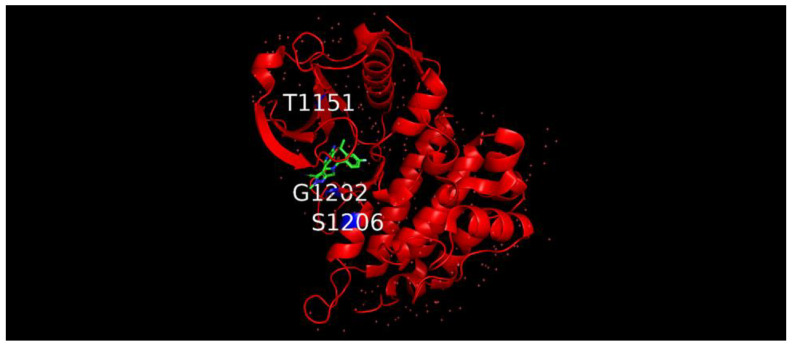
Crystallographic structure of ALK protein (red) in complex with PF-06463922 (lorlatinib), the structure of which is in green. The position of the three mutated residues is highlighted in blue (PDB accession number: 4CLI).

To better visualize the alterations in the protein tertiary structure, they were located in the ALK crystallographic structure in complex with lorlatinib. The location of the three altered residues is in close proximity to the binding pocket of the drug (Figure 3). 

Although further experimental studies are needed, it could be argued that the concomitant alterations disrupt the interaction with lorlatinib, leading to therapeutic resistance. 

In addition, Furuta et al. [29] recently reported an analysis on a group of 21 ALK-rearranged patients submitted to rebiopsy after ALK-TKI failure; the crucial role of genetic re-profiling together with a computational simulation model was highlighted in order to predict the ALK-TKI resistance mechanisms. 

In conclusion, this case emphasizes the need to retest patients during the disease course and at the time of progression on ALK-TKIs therapy. In particular, the need to use NGS sequencing techniques, with a multigene panel at high depth of coverage, is highlighted. In addition to tissue biopsy, blood-based molecular profiling, applied in this clinical setting, could provide further information about novel and complex acquired resistance mechanisms to ALK-inhibitors, particularly to lorlatinib. Such an approach could be crucial, favoring the best use of drugs and a tailored treatment strategy so as to improve the clinical outcome of these patients.

## Data Availability

Data are available upon reasonable request.
